# Automated Detection and Measurement of Isolated Retinal Arterioles by a Combination of Edge Enhancement and Cost Analysis

**DOI:** 10.1371/journal.pone.0091791

**Published:** 2014-03-13

**Authors:** José A. Fernández, Peter Bankhead, Huiyu Zhou, J. Graham McGeown, Tim M. Curtis

**Affiliations:** 1 Centre for Experimental Medicine, The Queen’s University of Belfast, Belfast, United Kingdom; 2 Centre for Cancer Research and Cell Biology, The Queen’s University of Belfast, Belfast, United Kingdom; 3 School of Electronics, Electrical Engineering and Computer Science, The Queen’s University of Belfast, Belfast, United Kingdom; Children’s Hospital Boston, United States of America

## Abstract

Pressure myography studies have played a crucial role in our understanding of vascular physiology and pathophysiology. Such studies depend upon the reliable measurement of changes in the diameter of isolated vessel segments over time. Although several software packages are available to carry out such measurements on small arteries and veins, no such software exists to study smaller vessels (<50 µm in diameter). We provide here a new, freely available open-source algorithm, MyoTracker, to measure and track changes in the diameter of small isolated retinal arterioles. The program has been developed as an ImageJ plug-in and uses a combination of cost analysis and edge enhancement to detect the vessel walls. In tests performed on a dataset of 102 images, automatic measurements were found to be comparable to those of manual ones. The program was also able to track both fast and slow constrictions and dilations during intraluminal pressure changes and following application of several drugs. Variability in automated measurements during analysis of videos and processing times were also investigated and are reported. MyoTracker is a new software to assist during pressure myography experiments on small isolated retinal arterioles. It provides fast and accurate measurements with low levels of noise and works with both individual images and videos. Although the program was developed to work with small arterioles, it is also capable of tracking the walls of other types of microvessels, including venules and capillaries. It also works well with larger arteries, and therefore may provide an alternative to other packages developed for larger vessels when its features are considered advantageous.

## Introduction

Pressure myography is widely used to monitor the responses of small arteries and veins to physical and chemical stimuli. This technique involves mounting a small isolated vessel segment between two glass cannulae and pressurising the vessel to an appropriate transmural pressure. By transferring the myograph to the stage of a microscope fitted with a video camera, changes in vasomotor activity can then be continuously imaged throughout the experiment. Pressure myography has been instrumental in our current understanding of the myogenic response (i.e. the intrinsic property of blood vessels to respond dynamically to changes in intraluminal pressure) and in characterising the pharmacological actions of drugs and other vasoactive compounds on the vascular system [1], [2]. Importantly, it has also provided crucial insights into the pathogenesis of vascular dysfunction in a range of different disease states, including, for example, hypertension, diabetes, obesity and stroke [3], [4], [5], [6], [7].

A key technical aspect of any myography-based study is the ability to accurately measure changes in vessel diameter over time. Traditional methods of manually measuring inner and outer vessel diameters remain popular [8], [9], [10], [11], but are time-consuming and prone to user bias [12]. Several automatic or semi-automatic algorithms have been developed to assist with these measurements. The SoftEdge Myocyte Cell Length Acquisition Module [13], [14], [15], [16] detects and tracks vessel walls within a couple of user-defined windows after a threshold value and a ‘crossing condition’ have been set. Another algorithm, developed by Kim et al. [17], involves the user defining a line perpendicular to the vessel and extending beyond the outer walls for intensity analysis at a high-contrast region of the vessel before carrying out Otsu’s thresholding. A more sophisticated algorithm, VesselTrack [18], [19], [20], detects the abluminal edges of the vessel walls in two small user-defined windows and tracks vessel movements in these regions using iterative regression. The adluminal edges are subsequently estimated from the detected outer values. Other packages that have also been used to study isolated vessels are MyoView (DMT) [21], [22] and the program Mary (Nihil, Lund, Sweden) [23]. In general, all of these programs rely on the use of thresholding methods which are well-suited to the analysis of arteries and veins where there is normally a high level of contrast between the vessel wall and other areas of the image.

Arterioles are small blood vessels that act as the major vascular resistance elements controlling blood flow from arteries to capillaries, and the properties of these vessels are of particular interest in improving our understanding of how local tissue perfusion and capillary pressure are regulated [24]. Although small vessels (<50 µm in diameter) are unsuitable for use with commercially available pressure myography systems, we have recently developed methods to carry out myography studies on isolated arterioles from the rat retina [25]. Like small arterioles from many other vascular beds, these vessels are surrounded by just a single layer of smooth muscle cells and consequently the vessel walls appear non-uniform, with no unambiguous change in contrast relative to the external background or intraluminal space [26]. This renders detection algorithms based solely on thresholding methods inadequate. We present here a free and open source ImageJ plug-in, MyoTracker, for the automatic tracking and measurement of the diameter of small isolated retinal arterioles in image files. The method used is based on a combination of edge enhancement and cost analysis. Thresholding is only used in the algorithm as a check on the detected borders. Our software can run on individual images and on stacks of thousands of images and is also amenable to the analysis of other vessel types, including larger arterial and arteriolar vessels, venules and capillaries.

## Methods

### Ethics Statement

All animal tissue was obtained by schedule 1 methods in accordance with the Animals (Scientific Procedures) Act 1986 and with the agreement of the Queen’s University of Belfast Animal Welfare and Ethical Review Body for which a specific project licence is not required.

### Vessel Preparation

Male Sprague-Dawley rats (8–12 weeks of age; 200–250 g) were euthanized using CO_2_ in accordance with UK legal and local institutional requirements. Retinas were placed in low Ca^2+^ Hanks’ solution containing (mmol/L): 140, NaCl; 6, KCl; 5, D-glucose; 0.1, CaCl_2_; 1.3, MgCl_2_; 10, HEPES (pH 7.4 with NaOH) and mechanically triturated using a Pasteur pipette. The tissue was pipetted into a recording bath mounted on an inverted microscope and isolated retinal arterioles, venules and capillaries (∼5–40 µm diameter) were identified as previously described [25], [27]. In one set of experiments, larger bovine arteries were used. Bovine eyes were obtained from a local abattoir and transported back to the laboratory in low Ca^2+^ Hanks’ solution at 1°C. The retinas were removed and the arteries mechanically isolated using the methods described above. All experiments were carried out at 37°C.

### Arteriolar Myography

The development of arteriolar myogenic tone was assessed using pressure myography, as described previously [28]. A tungsten wire slip (75×2000 µm) was laid on the arteriole, anchoring and occluding one end. The vessel was then superfused with Ca^2+^-free Hanks’ solution containing (mmol/L): 140, NaCl; 6, KCl; 5, D-glucose; 1.3, MgCl_2_; 10, HEPES (pH 7.4 with NaOH) at 37°C. The open end was cannulated using a glass micropipette (tip diameters 3–10 µm) filled with Ca^2+^-free medium, using a patch electrode holder and micromanipulator.

Following introduction of the pipette, the vessel was superfused with Hanks’ solution containing 2 mmol/L Ca^2+^ (added to the above solution as CaCl_2_) for 10–15 min, allowing the pipette to seal to the inner vessel wall. Intraluminal pressure was regulated by changing the height of a fluid reservoir connected to the inflow cannula and monitored using a pressure transducer. Individual vessels were viewed under a 20x, NA 0.4 objective and images (saved as BMP images of 1280×1024 pixels; 8-bit; 1.2 MB) captured at a rate of 140 images per minute using a MCN-B013-U USB camera. Acquisition was carried out using custom software implemented in Delphi. To convert pixels to microns, 102 pixels were found to be equivalent to 27 microns.

In separate experiments, vasoconstrictor responses of isolated, non-pressurised, retinal arterioles to 10 mM caffeine or 10 nM endothelin-1 (Et-1) were imaged using the same videomicroscopy set-up. Drugs were delivered via a gravity-fed multi-channel perfusion manifold connected to a single outlet needle (350 µm in diameter) that was positioned adjacent to the vessel of interest.

### Explanation of the Algorithm

**Figure 1:**
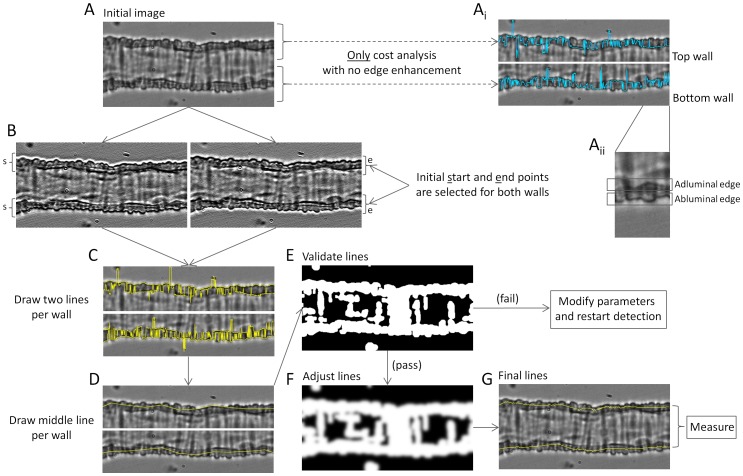
Description of the algorithm. A. Image of retinal arteriole under no pressure conditions and at 37°C. A_i_. Top and bottom walls from the arteriole. The blue lines superimposed are drawn by applying only cost analysis with no edge enhancement. Initial start and end points are used here as part of the cost function detection as shown below in B. A_ii_. Inset zoomed from the bottom wall in A_i_ illustrating adluminal and abluminal edges. B. Left and right panels correspond to images obtained from the vessel in A after convolving with 2 different kernels. Left image shows more pronounced top adluminal and bottom abluminal edges. Right image shows more pronounced top abluminal and bottom adluminal edges. Initial start (s) and end (e) points are also marked in the images. C. Top and bottom walls as in A. The 4 yellow lines superimposed correspond to the detected abluminal and adluminal edges in both walls. D. Estimated mid-wall lines (in yellow) are shown superimposed on the walls of the vessel. E. Binary image used for validation of the middle lines. If the validation fails, parameters are modified (as shown in Fig. 2) and detection is restarted. F. Modified binary image used to adjust the middle lines. This is obtained by applying a Gaussian filter and dilation on the image in E. G. The final lines obtained are superimposed on the vessel in yellow. These are the lines used to measure the diameter of the vessel.



Figure 1 illustrates the different steps used by MyoTracker to measure the diameter of an isolated retinal arteriole (Fig. 1 A). The software automatically detects the walls of the vessel, draws two lines along their centres, and measures the overall diameter as the mean of the vertical distances between these lines, at approximately one pixel intervals. A cost function was developed to assist with the drawing of the lines. This function balances out two main constraining factors: (a) the distance between the pixel being drawn and initially estimated points at the start and end of the vessel, and (b) the intensity changes (from darker to lighter values) along the vertical line where the pixel is located. More specifically, the cost function can be written as:
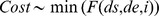
where 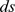 is defined as the distance of a pixel to a starting point and 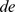 as the distance to an end point (i.e. distances to the lowest intensity points at the start and end of the vessel over five columns), and 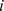 is the intensity of the pixel normalised by the average intensity over a number of pixels above and below it vertically. The function F is defined as follows:
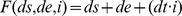
with:

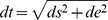



The advantages of using this cost function are (1) its efficiency of processing, (2) it achieves global optimisation using all the pixels on the vessel, and (3) it is sufficiently flexible, in that it only depends on the resulting terminal points and the intensity of the pixels. Given the high variability in intensity values observed along the walls of these small vessels, however, using the cost function alone usually gave ‘noisy’ lines (i.e. with many jumps between pixels at different intensities close to each other). This can be seen in the top right panels in Figure 1 (Fig. 1 A_i_, blue lines). To improve on this, several processing steps are carried out:

#### Step 1

Two filters are used to enhance the adluminal and abluminal edges of the vessel walls (Fig. 1 A_ii_). The first filter, with kernel [3, 1, –3], is used to enhance the adluminal edge of the top wall and the abluminal edge of the bottom wall (Fig. 1 B, left image), while the second filter with kernel [–3, 1, 3] is used to enhance the abluminal edge of the top wall and the adluminal edge of the bottom wall (Fig. 1 B, right image; see Fig. S1 for an explanation of the methodology followed to choose these kernel values). These filters provide a new vessel image with four enhanced edges, two on each wall, which facilitate the subsequent use of the cost function.

#### Step 2

Start and end points are automatically estimated by finding the darkest regions at both ends of the vessel (averaging over five columns; Fig. 1 B). Using these start and end points as initial parameters, the cost function is then used to draw lines along the four enhanced edges obtained in step 1, giving two lines per wall (Fig. 1 C, yellow lines).

#### Step 3

Lines are drawn along the centre of each wall using the four lines detected in step 2. To do this, a number of points per wall (dependent on the length of the vessel under analysis) are selected along the length of the vessel and the averages between the two lines corresponding to each wall at those points calculated. Joining these averaged values provides two new lines, corresponding to the detected vessel walls (Fig. 1 D).

#### Step 4

Validation of the two lines obtained in step 3 is carried out with the assistance of a binary image. The binary image is obtained using an initial Gaussian filter (with sigma value of 2), before applying an automated threshold determined using Otsu’s method [29], dilating the resulting image twice with a 3×3 kernel and filling holes, both in the normal and the inverted images (Fig. 1 E). As can be seen in Figure 1, the image obtained by applying thresholding methods is not good enough to unambiguously determine the centre of the vessel walls with certainty, as several other structures also appear as part of the mask (white regions of the binary image). This image, however, is good enough to check whether the already obtained lines fall within the mask, as an additional validation.

#### Step 5

After the lines are validated (i.e. they were contained in the mask), they are further adjusted using a modified version of the binary image from step 4. This modified image is obtained by further dilating the mask and by applying a Gaussian filter to the image (Fig. 1 F). Each pixel in the two estimated lines is then shifted up or down until the lightest intensity values present in the new smoothed binary image are reached, bringing them closer to the centre of the wall in those cases where they might have been drawn very close to the edge.

#### Step 6

The final lines are used to measure the diameter of the vessel (Fig. 1 G). This final measurement is obtained by averaging the vertical distances between the two lines over all pixels from left to right.

To estimate the computational complexity of the algorithm, the image to be processed is assumed to have a size *M* * *N*, and a kernel with size (2*R*+1) * (2*R*+1). The computational complexity can then be obtained for each step of the algorithm as follows: (1) Step 1: *O*(*MNR* ^2^). (2) Step 2: *O*(*MN*). (3) Step 3: *O*(*S*) (*S* – sample points along the vessel). (4) Step 4: *O*(*MN*(2*9+*P* ^2^)+*L* ^4^) (*P* is the kernel size of the Gaussian filter, and *L* is the number of gray levels). (5) Step 5: *O*(*MNP* ^2^). (6) Step 6: *O*(*Q*) (*Q* is the number of pixels on one line). Finally, the total computational complexity of the proposed algorithm can be expressed as: *O*(*MN*(*R* ^2^+ *P* ^2^+19)+*L* ^4^+*S*+*Q*).

The algorithm implements automatic restarting and fitting of parameters when needed (for steps 2 to 4). Briefly, when the lines drawn in step 3 do not pass the validation test (step 4), several parameters are adjusted (up to a maximum value) to provide a better fit of the data and the detection is restarted (back to step 2).

**Figure 2:**
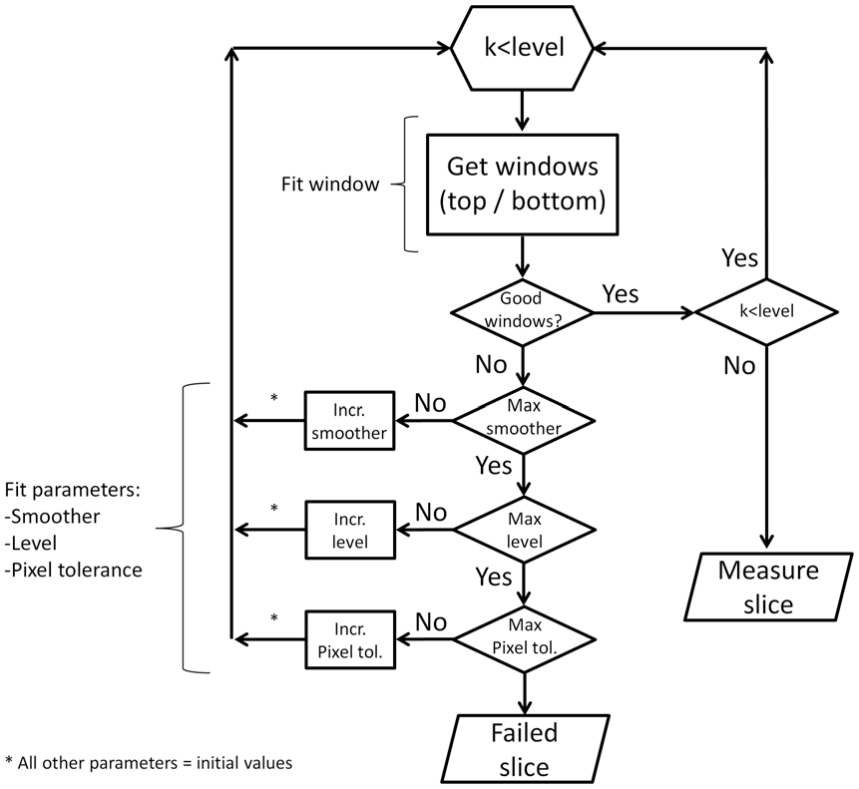
Flowchart describing the fitting of the different parameters. Iterative changes are made to the values of several parameters during the running of the program. Some of these values are increased in a fixed order (*smoother* first, *level* second and *pixel tolerance* third) up to a maximum value, giving different iterations of the algorithm. Good detection windows are searched at each iteration by modifying the parameter *window* within its range of values. Given that the parameter *level* works by dividing the original image into narrower images, it requires some added functionality. Increasing the level from 1 to 2, for example, duplicates the number of images on which to carry out the analysis. Instead of the initial full length image, the algorithm now runs on 2 smaller images (of height equal to the original image) independently, each corresponding to the left and right halves of the initial image. The variable *k* is used to count which of the sub-images is being analysed.

Figure 2 gives a lower level illustration of the fitting process. The following parameters are fitted by the program:

#### Window

Divides the image into increasingly narrower horizontal subsections of width equal to that of the original image (ranging from 2 to 6).

#### Level

Divides the image into increasingly narrower vertical subsections of height equal to that of the original image (ranging from 1 to 5).

#### Smoother

Determines the number of points to be used in the drawing of the central lines at step 3. The initial number of points depends on the length of the vessel (a default distance between points of 25 pixels is initially set, with the maximum number of points limited to 40).

#### Pixel tolerance

Determines the maximum number of pixels outside the binary mask that can be allowed during validation at step 4 (given as a percentage of the overall length of the vessel; ranging from 1 to 50%).

The goal of these parameters is to increase the probability of finding subsections within the image where good lines can be detected. If the values for all the parameters are exhausted and no good lines are found, the image fails and no final measurement is provided.

### Presentation of the Results

MyoTracker runs independently on each image/slice. After the last slice is analysed, the program optionally displays the detected vessel walls as an overlay on the image for verification if desired, and provides diameter values in a results table and/or plot. A manual is also available, which describes the outputs and adjustable parameters used by the algorithm (File S1). However, fine-tuning of parameters is normally not necessary. All results presented in this paper were obtained using the default parameter values, unless otherwise stated.

### Testing the Program

An ImageJ macro was developed for manual vessel analysis to assist validation of the MyoTracker algorithm. The macro allowed for tests to be carried out over a period of time so that tiredness did not have a negative impact on the accuracy of the measurements. The diameters of retinal arterioles from 102 test images were manually measured by 3 different researchers. For this, each user was required to draw two lines (using the segmented lines tool in ImageJ) per image along the centre of both top and bottom walls and the macro determined the distance between them. These measurements were subsequently compared to the automatic measurements obtained by MyoTracker (ImageJ version 1.48a).

### Processing Times

Another macro was developed to assist with the analysis of the performance of the algorithm, i.e. the processing time required by the algorithm to analyse both individual images and videos containing different numbers of slices. The macro was run on all 102 images from the dataset and on several videos, and averaged times (over 10 different runs for each image and video) were obtained on a PC with an Intel® Core™ i7-2600 and 16 GB of RAM.

### Statistics

To assess the agreement between automatic and manual measurements, the standard deviation of the measurement error (calculated as the difference between automatic and manual values) was determined as previously described [30]. Bland-Altman plots were also constructed as an additional method of assessment [31], [32], with the manual measurements used as the reference or ‘gold standard’ method [33]. The plots were performed using MedCalc for Windows, version 12.7.5.0 (MedCalc Software, Ostend, Belgium). Summary data is expressed as means ± SD. In all cases, the accepted significance level was set at 0.05.

## Results

### Comparison between Manual and Automatic Measurements

**Figure 3:**
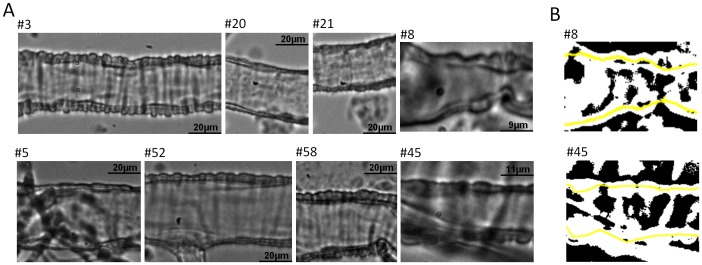
Examples of images taken from the dataset used for analysis. A. Images of 8 retinal arterioles illustrating the diversity we can expect from these kinds of images. B. Binary images made from panels #8 (top) and #45 (bottom) shown on A. The lines estimated by the algorithm are superimposed on these in yellow.

Figure 3 shows a sample of 8 images taken from a full dataset of 102 retinal arteriole images (Fig. 3 A). This subset illustrates the different widths, lengths, positions, focus, contrasts and intensities considered during testing. Each of these factors contributed to the overall complexity of the analysis. In Figure 3 there are also two binary images obtained from panels #8 and #45 (Fig. 3 B, top and bottom images, respectively). These give an indication of the structures that are detected when carrying out threshold analysis alone. The lines detected by MyoTracker are superimposed on the binary images (Fig. 3 B, top and bottom, yellow lines).

Manual and automatic measurements were carried out in the full dataset containing 102 images. The results are presented in Table 1. The mean values for manual and automatic measurements were 26.8±8.7 and 26.8±8.8 µm, respectively. The mean of the measurement error for the MyoTracker software versus the manual measurements was 0.02 µm, whereas its standard deviation was 0.52 µm. This value was lower than any of the standard deviations obtained by comparing each of the 3 manual measurements with the mean of the other 2 in each case (0.9, 0.88 and 0.76 µm).

**Table 1:** Manual and automatic measurements on dataset.

Image	Manual a	Automatic b		Manual	Automatic		Manual	Automatic
#1	27±0.2	28	#35	31±0.2	31	#69	36±0.2	36
#2	20±1.2	21	#36	35±0.9	35	#70	35±0.2	35
#3	28±0.4	28	#37	40±0.6	39	#71	35±0.2	34
#4	34±0.3	34	#38	40±0.4	40	#72	25±0.3	25
#5	34±0.3	34	#39	31±2	32	#73	16±0.1	16
#6	33±0.1	32	#40	36±1.8	36	#74	28±0.8	27
#7	34±0.6	34	#41	34±0.9	34	#75	12±0.4	11
#8	14±1.1	13	#42	26±1	25	#76	14±0.4	14
#9	16±0.6	16	#43	31±0.6	30	#77	17±0.9	17
#10	22±2.7	22	#44	34±0.2	34	#78	19±0.4	19
#11	32±1.3	32	#45	18±1.7	17	#79	19±0.3	19
#12	30±1.6	29	#46	25±0.6	26	#80	20±0.6	20
#13	34±0.7	34	#47	28±0.5	27	#81	16±0.3	15
#14	28±2.3	28	#48	33±0.4	33	#82	16±0.3	16
#15	37±0.1	36	#49	32±0.2	32	#83	16±0.4	16
#16	23±0.2	22	#50	26±0.2	27	#84	17±0.6	17
#17	27±0.6	28	#51	36±0.8	36	#85	26±1.1	25
#18	28±0.6	29	#52	36±0.5	37	#86	27±0.5	26
#19	27±0.4	27	#53	37±0.3	37	#87	8±0.6	8
#20	30±0.6	30	#54	36±1.1	36	#88	8±0.3	7
#21	26±0.1	26	#55	31±0.3	32	#89	7±0.2	8
#22	32±0.1	33	#56	26±1.1	26	#90	9±0.6	9
#23	32±0.6	33	#57	26±1.6	26	#91	9±0.6	9
#24	34±1.4	34	#58	32±0.8	32	#92	11±0.4	11
#25	35±0.6	36	#59	37±0.3	38	#93	12±0.2	12
#26	12±0.5	12	#60	23±1.4	23	#94	12±0.2	12
#27	13±0.5	12	#61	24±0.2	24	#95	13±0.5	12
#28	29±0.9	29	#62	34±0.3	34	#96	36±0.3	36
#29	39±0.8	39	#63	29±0.7	30	#97	34±0.2	34
#30	39±0.5	39	#64	35±1	35	#98	33±0.7	33
#31	32±0.1	32	#65	32±0.4	33	#99	31±0.4	31
#32	32±0.3	32	#66	34±0.3	34	#100	32±0.4	32
#33	34±0.3	34	#67	32±0.1	33	#101	25±0.2	25
#34	22±0.3	23	#68	26±0.2	26	#102	31±0.4	30

a Averaged values (mean ± SD) from measurements (in µm) taken by 3 different users.

b Automatically measured values (in µm) taken by the algorithm with default parameter values (i.e. *level* = 1, *preferred smoothing spacing* = 25, *pixel tolerance* = 1, and no additional processing).

**Figure 4:**
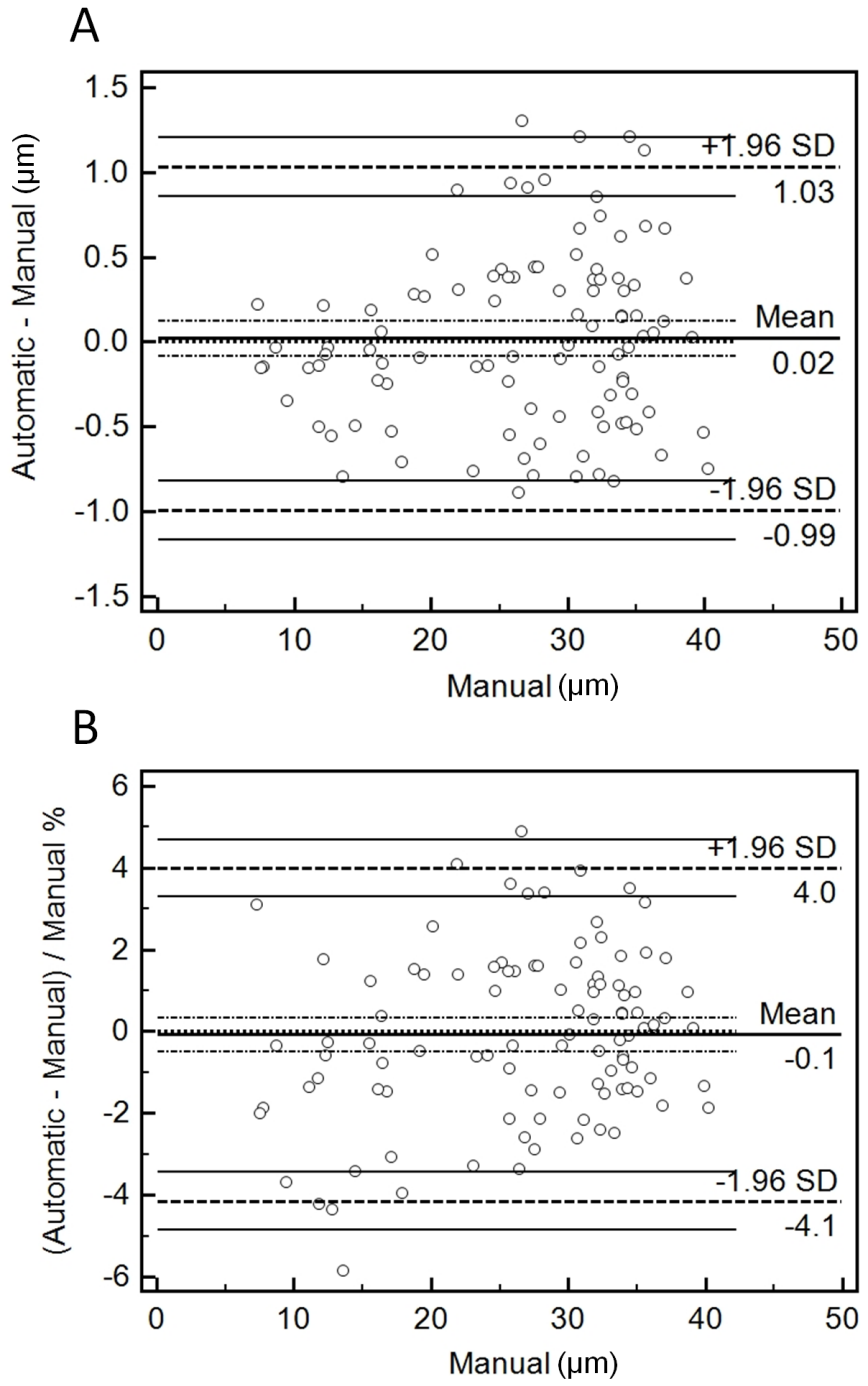
Bland-Altman plots. A. Plot of the difference between automatic and manual measurements against the manual ones (used as ‘gold standard’). The Mean line is indicated in the middle of the plot, giving the overall mean of the differences. Dashed lines are shown immediately on top and bottom of the Mean line indicating the 95% confidence intervals. Another dashed line marks also the equality line (difference of 0 between automatic and manual). Two further dashed lines are also drawn at the top and bottom of the plot indicating the ±1.96 SD limits of agreement, with their respective 95% confidence intervals. Each circle indicates an individual raw difference. B. Same as in A, but in this case percentage values are plotted instead (relative to the width of the vessels).

Bland-Altman plots were also used to assess the agreement between measurements. Figure 4 shows these plots for both raw differences (Fig. 4 A) and also differences relative to the width of the vessels (Fig. 4 B). As can be seen, in almost all images errors were within ±1.5 µm, or ±5% of the width of the vessels. The only image with an error higher than 5% was image #8 (error = ∼6%; Fig. 4 B). This value, however, was close to the best relative error seen when comparing all the manual measurements with each other for this image (5.33, 10.01 and 15.33%). Both plots show that the mean of the errors (solid lines in Fig. 4 A and B) was within the confidence intervals of an error of 0, indicating that the automatic measurements were not significantly different from the manual ones. The distribution of the measurement error data also showed that there was no systematic bias in relation to vessel width.

### Automatic Measurements on Videos

To investigate the variability observed in the automatic measurements during video recordings, MyoTracker was used to measure the diameter of retinal arterioles in recordings containing around 100 images each. Only vessels under steady-state conditions and with no visually observable constrictions or dilations were used for this analysis. The values obtained in this analysis had standard deviations ranging from 0.03 to 0.15 µm (with a mean of 0.08 µm, n = 5). In all cases the variability remained below 1% of the width of the vessels (ranging from 0.24 to 0.66%, with a mean of 0.35%).

### Analysis of Vessel Movements

**Figure 5:**
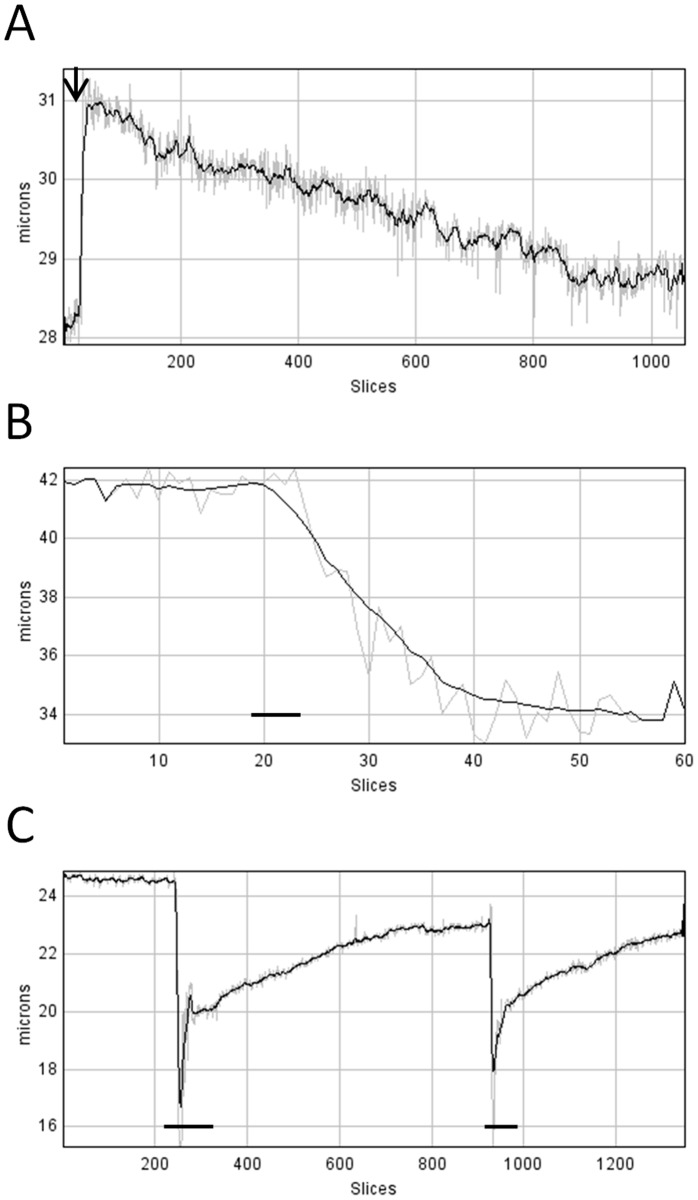
Diameter changes during vessel movements. A. Time course of dilation of a cannulated retinal arteriole to pressurisation from 0 to 40(indicated by a black arrow on top of the plot) followed by myogenic tone development over 7 minutes. The analysis was carried out using the *preprocessing* parameter to facilitate detection of the vessel walls and *inclination correction* to avoid introducing errors in the measurement of the diameters caused by the inclination of the vessel (see Manual of the software, File S1). B. Time course of constriction of a retinal arteriole to application of endothelin-1 (10 nM, horizontal black line). C. Time course of fast constrictions and slow dilations of a retinal arteriole caused by successive applications of caffeine (10 mM, horizontal black lines) over a period of ∼10 minutes. In all plots, a running average (black line) with a bin of 11 slices was superimposed on top of the raw diameters (grey line), as described in the software’s manual (File S1).

Tests were carried out on videos showing changes in the diameter of arterioles, both constricting and dilating to different external stimuli. The top panel of Figure 5 shows the time course of initial dilation of an isolated and cannulated retinal arteriole during pressurisation from 0 to 40 mmHg (black arrow) and the subsequent development of myogenic response over a period of 7 minutes (Fig. 5 A). The vessel dilated from 28.2 to 30.9 µm (steady-state values) at a rate of over 6 µm per second before constricting by about 2 µm (Fig. 5 A). The time course of the constriction of a different arteriole to application of the vasoconstrictor peptide, endothelin-1 (10 nM), is shown in the middle panel (Fig. 5 B, black line). In this case, the diameter of the vessel was reduced from 41.7 to 34.1 µm (steady-state values) at an approximate rate of 0.57 µm per second. Finally, the time course of two fast constrictions and subsequent slow dilations to caffeine (10 mM) is shown in the panel at the bottom (Fig. 5 C, black lines). In the first application of caffeine, the diameter of the vessel was reduced from 24.6 to 15.3 µm at a rate of 2.17 µm per second. In all cases the program was able to track the changes.

### Measuring other Kinds of Vessels

**Figure 6:**
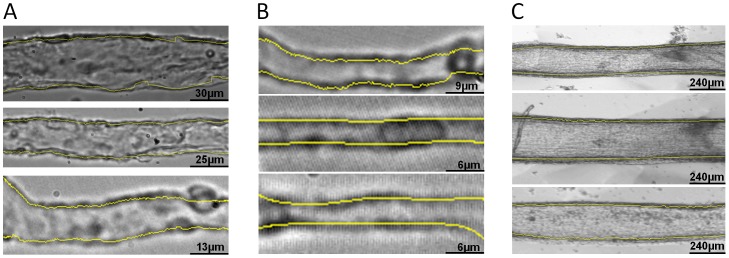
Different vessels tested with MyoTracker. Venules (A) and capillaries (B) from rat retina, and bigger arteries (C) from bovine retinal tissues used to test the algorithm. The lines detected by MyoTracker are shown superimposed in yellow on top of the vessels. Spline interpolation was used to improve the detection of the walls in all vessels in section A. The detection of 2 of the capillaries also required the assistance of some additional parameters from the program (see the manual of the software, File S1). To detect the walls in the middle and bottom panels in section B, *preprocessing* was checked. The other parameters were as follows: *Level* = 1, *Preferred smoothing spacing* = 25, *Pixel tolerance* = 100, *Derivative* = 2, *Light correction* = 6 (with the flags *light correction* and *Use spline interpolation* checked). The detection of the walls in the arteries was carried out with *Preferred smoothing spacing* = 100 (the other parameters had default values).

Apart from arterioles, other types of retinal vessels were also tested using the MyoTracker software. We initially tested isolated retinal venules and capillaries where the adluminal and abluminal edges of the vessel walls were less obvious due to the absence of smooth muscle cells. Figure 6 shows examples of isolated retinal venules (Fig. 6 A) and capillaries (Fig. 6 B) of different sizes. The detected lines are shown superimposed on top of each of the vessels (Fig. 6 A and B, yellow lines). As can be seen, the program is capable of detecting the walls of these vessels, generally ignoring other elements present in and around the vessels, such as blood cells. We also tested the program with larger bovine arteries (Fig. 6 C). As can be seen, Myotracker was also able to track the walls of these vessels (Fig. 6 C, yellow lines).

### Processing Times

The processing times required by MyoTracker to run on each of the 102 images contained in the dataset were recorded and appear in Table 2. Each of the times represents the average of 10 independent runs of the program for each image. The average processing time was 19 ms, ranging from 7.7 to 100 ms. The variability observed between the times for the 10 runs on the same image was always below 6 ms (with mean standard deviation of 1.7 ms). In this dataset, there was no correlation between mean times and the size of the images (R = 0.35). It is expected, however, that processing time will increase with complex images, where the algorithm might need to iterate over some of the fitting parameters to find appropriate values in the analysis (see Methods). Analysis carried out on ‘image stacks’ (equivalent to video files) with increasing number of images showed that, in general, processing time grows linearly with the number of images (Table 3).

**Table 2:** Processing times for images on dataset.

Image	Times a	Size b		Times	Size		Times	Size
#1	12±2.6	41013	#35	16±0.5	47430	#69	19±0.5	87438
#2	14±5	37824	#36	14±0.5	60078	#70	17±0.5	79781
#3	19±0.4	95484	#37	20±0.7	97290	#71	20±0.7	94119
#4	60±0.5	98241	#38	22±0.7	96317	#72	11±0.4	40076
#5	16±0.3	63612	#39	14±5.7	47616	#73	8±2.5	12168
#6	12±4	42180	#40	12±4.1	21285	#74	17±2.8	65987
#7	11±3.9	25521	#41	9±0.5	30351	#75	8±2.6	9040
#8	10±1.6	10710	#42	9±2.9	19360	#76	15±4.3	23925
#9	28±4.4	24600	#43	10±2.1	20768	#77	28±0.5	50370
#10	10±0.6	35260	#44	12±3.3	30624	#78	15±5.6	48762
#11	12±4.2	37848	#45	25±0.8	24660	#79	11±2.1	25536
#12	100±2.9	58292	#46	23±0.3	26344	#80	15±5.2	43952
#13	14±0.5	58292	#47	12±4.1	35600	#81	17±3.1	65349
#14	99±0.8	58292	#48	12±3.8	40228	#82	18±3	33264
#15	17±0.5	80769	#49	12±0.3	37572	#83	13±3.8	37488
#16	34±0.3	33115	#50	21±0.5	93100	#84	15±5.5	38556
#17	15±4.7	48051	#51	65±0.9	66768	#85	16±4.4	49010
#18	32±0.4	29925	#52	16±0.6	66768	#86	13±4.1	41237
#19	52±0.6	38304	#53	16±0.5	73402	#87	9±2	8509
#20	14±0.7	48600	#54	75±0.7	73188	#88	12±3.2	17043
#21	13±0.6	48600	#55	16±0.5	70620	#89	12±2.1	21627
#22	14±4.6	31325	#56	11±4	32922	#90	9±0.3	11786
#23	15±5.5	31325	#57	13±4.7	42180	#91	12±1.5	17748
#24	9±1.6	9288	#58	49±0.7	49284	#92	13±3.1	26623
#25	11±3.3	22833	#59	73±0.5	57276	#93	11±3.8	23700
#26	8±2.2	5856	#60	14±0.7	64845	#94	13±3.1	30302
#27	10±1.5	10464	#61	13±0.4	54450	#95	15±5.2	32870
#28	13±0.4	34560	#62	68±0.7	70620	#96	13±2.1	34069
#29	14±0.7	64581	#63	9±3.2	20768	#97	15±4.4	45310
#30	16±3.7	62282	#64	16±3.7	60078	#98	8±2.5	17800
#31	12±0.4	43400	#65	11±0.4	43400	#99	16±0.4	74983
#32	11±0.5	43400	#66	11±0.4	46600	#100	10±0.7	32494
#33	11±0.7	46600	#67	13±4.6	31325	#101	15±0.7	63918
#34	10±0.3	39744	#68	12±2.6	28864	#102	15±4.2	51336

a Averaged (mean ± SD) processing times (in ms) required to automatically analyse each image from 10 independent runs.

b Size (length×width) of the image analysed (in pixels).

**Table 3:** Processing times for videos.

Number of images^a^	Processing times for videos^b^
2	13±0.9
10	42±6.9
50	165±11
100	308±9.1
500	1510±20
1000	3016±49
5000	15175±102

a Number of slices included in the video under analysis. Each image was a copy of image #1 (of size 279×147 pixels) from the dataset in Table 2.

b Averaged (mean ± SD) processing times (in ms) required to automatically analyse each video from 10 independent runs.

## Discussion

Previous studies concerning myogenic mechanisms in small isolated arterioles (<50 µm in diameter) have, at least in part, been hampered by the lack of appropriate software for accurately automating the analysis of vessel diameter. We have presented here a software package that facilitates this analysis. Measuring and tracking the diameter of small arterioles during pressure myography experiments is complicated by the complexity of the images obtained. The image dataset used in this study contained a large diversity of images with different levels of complexity. Using this dataset MyoTracker performed well, providing automatic measurements that were comparable to those obtained manually by three independent researchers (Figs. 3 and 4; Table 1).

An advantage of our software compared to other programs is that MyoTracker works in a fully automated way, with no need for the user to take any manual measurements, define any initial windows or mark the walls of the vessel for analysis prior to running the program. Moreover, this program measures and tracks the diameter of the vessel over the full vessel segment present in the image, as opposed to carrying out the measurements on a small window of interest. Tracking of vessel diameters was tested with videos of images taken during experiments and the program was able to track both fast and slow changes along the full segments of the vessels and over the full duration of the experiments (Fig. 5). Given these advantages, this software may also be of interest for use with larger arteries and veins and we have shown that MyoTracker works well with such vessels (Fig. 6). There is also increasing evidence that abluminal pericytes on small post-capillary venules and capillaries are capable of constricting to modulate vessel diameter, and thus, may contribute to the control of local tissue blood flow and capillary pressures [34], [35], [36], [37]. We have shown that MyoTracker is also able to detect the borders of these vessels and therefore may be useful in studying control mechanisms throughout the microvascular system (Fig. 6).

Despite its advantages, MyoTracker does also have some limitations. Firstly, it requires the vessel to be orientated horizontally within the field of view and that both ends of the vessel reach the ends of the image. Although images can be cropped and rotated to accomplish this, in our experience, vessels can occasionally change their angle during experimentation (e.g. during pressurisation), adding a small inclination-dependent error to the measurements in some, but not all slices. As explained in the user manual (File S1), additional parameters have been incorporated into the software to help circumvent this problem. Briefly, instead of calculating the distance between the vessel walls vertically, in videos where this problem exists the shortest distance between the detected lines can be calculated on a pixel-by-pixel basis using a distance transform. Another limitation is that MyoTracker yields diameter data from the centre of the vessel walls rather than the adluminal or abluminal edges. This design choice was motivated by the need to find an alternative detection method not dependent on thresholding algorithms. Although cost analysis and edge enhancement provided such an alternative, it was necessary to combine the adluminal and abluminal detected lines (i.e. use them to correct each other) to add consistency and reliability to the detection algorithm. However, given that the main interest in many experiments is the tracking of vessel diameters with the goal of identifying relative changes (i.e. constriction or dilations) over time, detecting the middle of the vessel walls can serve this purpose.

To conclude, we present here a program to facilitate the measurement and tracking of the diameter of small isolated retinal arterioles during myography experiments. Our program has been tested with images taken from experiments on isolated retinal arterioles, venules and capillaries of different sizes. However, it is also likely to work on vessels from other microvasculature tissues, as they all present similar detection problems. MyoTracker may also be used to study larger vessels when its features are considered advantageous when compared to other software packages.

### Algorithm Availability

The plug-in implementation is included as supporting information, together with a user manual (File S1). The datasets of images used for this study are also provided (Dataset S1 and S2).

## Supporting Information

Figure S1 **Measurement errors for different parameters in the algorithm.** The error is shown as the SD of the difference between automatic and manual measurements in each case. The automatic values were obtained by the algorithm with either, only cost function and no further correction, or cost function and 6 different corrections: no kernel (using the original image with fitting parameters as outlined in Fig. 2 but without edge enhancement), k0 (using edge enhancement with kernels top = [−1, 0, 1] and bottom = [1,0,−1]), k1 (top = [−1, 1, 1] and bottom = [1,1,−1]), k2 (top = [−2, 1, 2] and bottom = [2,1,−2]), k3 (top = [−3, 1, 3] and bottom = [3,1,−3]), and k4 (top = [−4, 1, 4] and bottom = [4,1,−4]). The measurements were carried out in 101 of the 102 images in the dataset (image #8 was left out of the analysis because the detection failed with no kernel and with k1). The plot shows a significant improvement (decrease) in the values of the measurement errors with increasing correction (P<0.001 between only cost function and the last 3 kernels, k2, k3 and k4), up to a point where no further significant improvement was detected (P<0.01 between k2 and k3; P>0.05 between k3 and k4). Thus, k3 was selected as the default kernel for the algorithm in this study. Significance was estimated using One-way Repeated Measures ANOVA with Newman-Keuls Multiple Comparison post-hoc test.
(TIF)

File S1 **MyoTracker’s code and manual.** The code folder contains both the source code (JAVA File) and the Executable Jar File for the plug-in. This allows the execution of the software within the FIJI (Image-J) environment, as explained in the manual. Copyright and GNU General Public License files are also included in this folder. The documentation folder includes the manual with instructions regarding the installation and use of the software.
(ZIP)

Dataset S1 **First set of 51 images of retinal arterioles used for the analysis.**
(ZIP)

Dataset S2 **Second set of 51 images of retinal arterioles used for the analysis.**
(ZIP)

## References

[1] Davis MJ, Hill MA (1999) Signaling mechanisms underlying the vascular myogenic response. Physiol Rev 79(2): 387–423. 10221985 10.1152/physrev.1999.79.2.387

[2] Davis MJ (2012) Perspective: physiological role(s) of the vascular myogenic response. Microcirculation 19(2): 99–114. 21895845 10.1111/j.1549-8719.2011.00131.x

[3] Howitt L, Sandow SL, Grayson TH, Ellis ZE, Morris MJ, et al (2011) Differential effects of diet-induced obesity on BKCa {beta}1-subunit expression and function in rat skeletal muscle arterioles and small cerebral arteries. Am J Physiol Heart Circ Physiol 301(1): H29–40. 21536854 10.1152/ajpheart.00134.2011

[4] Hein TW, Potts LB, Xu W, Yuen JZ, Kuo L (2012) Temporal development of retinal arteriolar endothelial dysfunction in porcine type 1 diabetes. Invest Ophthalmol Vis Sci 53(13): 7943–9. 23139282 10.1167/iovs.12-11005 PMC3513275

[5] Burke M, Pabbidi MR, Farley J, Roman RJ (2013) Molecular mechanisms of renal blood flow autoregulation. Curr Vasc Pharmacol (in press) [Epub ahead of print]. 10.2174/15701611113116660149 PMC4416696 24066938

[6] Izzard AS, Heagerty AM (2013) Myogenic properties of brain and cardiac vessels and their relation to disease. Curr Vasc Pharmacol (in press) [Epub ahead of print]. 10.2174/15701611113116660150 24066936

[7] Palomares SM, Cipolla MJ (2013) Myogenic tone as a therapeutic target for ischemic stroke. Curr Vasc Pharmacol (in press) [Epub ahead of print]. 10.2174/15701611113116660155 24066932

[8] Czikora Á, Lizanecz E, Bakó P, Rutkai I, Ruzsnavszky F, et al (2012) Structure-activity relationships of vanilloid receptor agonists for arteriolar TRPV1. Br J Pharmacol 165(6): 1801–12. 21883148 10.1111/j.1476-5381.2011.01645.x PMC3372831

[9] Fike CD, Kaplowitz M, Zhang Y, Dantuma M, Madden JA (2012) Effect of a phosphodiesterase 5 inhibitor on pulmonary and cerebral arteries of newborn piglets with chronic hypoxia-induced pulmonary hypertension. Neonatology 101(1): 28–39. 21791937 10.1159/000326270 PMC3151003

[10] Chan SL, Sweet JG, Cipolla MJ (2013) Treatment for cerebral small vessel disease: effect of relaxin on the function and structure of cerebral parenchymal arterioles during hypertension. FASEB J (in press) [Epub ahead of print]. 10.1096/fj.13-230797 PMC4046185 23783073

[11] Tanano I, Nagaoka T, Omae T, Ishibazawa A, Kamiya T, et al (2013) Dilation of porcine retinal arterioles to cilostazol: roles of eNOS phosphorylation via cAMP/protein kinase A and AMP-activated protein kinase and potassium channels. Invest Ophthalmol Vis Sci 54(2): 1443–9. 23341020 10.1167/iovs.12-10115

[12] Simons MA, Bastian BV, Bray BE, Dedrickson DR (1987) Comparison of observer and videodensitometric measurements of simulated coronary artery stenoses. Invest Radiol 22: 562–8. 3305413 10.1097/00004424-198707000-00006

[13] Gokina NI, Osol G (2002) Actin cytoskeletal modulation of pressure-induced depolarization and Ca^2+^ influx in cerebral arteries. Am J Physiol Heart Circ Physiol 282(4): H1410–20. 11893578 10.1152/ajpheart.00441.2001

[14] Zhong XZ, Harhun MI, Olesen SP, Ohya S, Moffatt JD, et al (2010) Participation of KCNQ (Kv7) potassium channels in myogenic control of cerebral arterial diameter. J Physiol 588(Pt 17): 3277–93. 10.1113/jphysiol.2010.192823 PMC2976022 20624791

[15] Hannah RM, Dunn KM, Bonev AD, Nelson MT (2011) Endothelial SK(Ca) and IK(Ca) channels regulate brain parenchymal arteriolar diameter and cortical cerebral blood flow. J Cereb Blood Flow Metab 31(5): 1175–86. 21179072 10.1038/jcbfm.2010.214 PMC3099631

[16] Dabertrand F, Nelson MT, Brayden JE (2012) Acidosis dilates brain parenchymal arterioles by conversion of calcium waves to sparks to activate BK channels. Circ Res 110(2): 285–94. 22095728 10.1161/CIRCRESAHA.111.258145 PMC3505882

[17] Kim S, Kong RL, Popel AS, Intaglietta M, Johnson PC (2006) A computer-based method for determination of the cell-free layer width in microcirculation. Microcirculation 13(3): 199–207. 16627362 10.1080/10739680600556878

[18] Davis MJ (2005) An improved, computer-based method to automatically track internal and external diameter of isolated microvessels. Microcirculation 12(4): 361–72. 16020082 10.1080/10739680590934772

[19] Davis MJ, Zawieja DC, Gashev AA (2006) Automated measurement of diameter and contraction waves of cannulated lymphatic microvessels. Lymphat Res Biol 4(1): 3–10. 16569200 10.1089/lrb.2006.4.3

[20] Nepiyushchikh ZV, Chakraborty S, Wang W, Davis MJ, Zawieja DC, et al (2011) Differential effects of myosin light chain kinase inhibition on contractility, force development and myosin lightchain 20 phosphorylation of rat cervical and thoracic duct lymphatics. J Physiol 589(Pt 22): 5415–29. 10.1113/jphysiol.2011.218446 PMC3240881 21930597

[21] Chan YC, Leung FP, Wong WT, Tian XY, Yung LM, et al (2010) Therapeutically relevant concentrations of raloxifene dilate pressurized rat resistance arteries via calcium-dependent endothelial nitric oxide synthase activation. Arterioscler Thromb Vasc Biol 30(5): 992–9. 20185791 10.1161/ATVBAHA.110.203935

[22] Chan YC, Leung FP, Tian XY, Yung LM, Lau CW, et al (2012) Raloxifene improves vascular reactivity in pressurized septal coronary arteries of ovariectomized hamsters fed cholesterol diet. Pharmacol Res 65(2): 182–8. 22005391 10.1016/j.phrs.2011.09.010

[23] Grände G, Nilsson E, Edvinsson L (2013) Comparison of responses to vasoactive drugs in human and rat cerebral arteries using myography andpressurized cerebral artery method. Cephalalgia 33(3): 152–9. 23197351 10.1177/0333102412468340

[24] Jackson WF (2000) Ion Channels and Vascular Tone. Hypertension 35(1 Pt 2): 173–8. 10.1161/01.hyp.35.1.173 PMC1382026 10642294

[25] McGahon MK, Dawicki JM, Arora A, Simpson DA, Gardiner TA, et al (2007) Kv1.5 is a major component underlying the A-type potassium current in retinal arteriolar smooth muscle. Am J Physiol Heart Circ Physiol 292(2): H1001–8. 17040965 10.1152/ajpheart.01003.2006 PMC2593469

[26] Scholfield CN, McGeown JG, Curtis TM (2007) Cellular physiology of retinal and choroidal arteriolar smooth muscle cells. Microcirculation 14: 11–24. 17365658 10.1080/10739680601072115

[27] Hinds K, Monaghan KP, Frolund B, McGeown JG, Curtis T (2013) GABAergic control of arteriolar diameter in the rat retina. Invest Ophthalmol Vis Sci (in press) [Epub ahead of print]. 10.1167/iovs.13-12362 24045989

[28] McGahon MK, Dash DP, Arora A, Wall N, Dawicki J, et al (2007) Diabetes downregulates large-conductance Ca^2+^-activated potassium beta 1 channel subunit in retinal arteriolar smooth muscle. Circ Res 100: 703–11. 17293477 10.1161/01.RES.0000260182.36481.c9 PMC2596350

[29] Otsu N (1979) A threshold selection method from gray-level histograms. IEEE Trans Syst Man Cybern 9: 62–6.

[30] Al-Diri B, Hunter A, Steel D (2009) An active contour model for segmenting and measuring retinal vessels. IEEE Trans Med Imaging 28(9): 1488–97. 19336294 10.1109/TMI.2009.2017941

[31] Bland JM, Altman DG (1986) Statistical methods for assessing agreement between two methods of clinical measurement. Lancet 1(8476): 307–10. 2868172

[32] Bland JM, Altman DG (1999) Measuring agreement in method comparison studies. Stat Methods Med Res 8(2): 135–60. 10501650 10.1177/096228029900800204

[33] Krouwer JS (2008) Why Bland-Altman plots should use X, not (Y+X)/2 when X is a reference method. Stat Med 27(5): 778–80. 17907247 10.1002/sim.3086

[34] Bandopadhyay R, Orte C, Lawrenson JG, Reid AR, De Silva S, et al (2001) Contractile proteins in pericytes at the blood-brain and blood-retinal barriers. J Neurocytol 30(1): 35–44. 11577244 10.1023/a:1011965307612

[35] Hughes S, Chan-Ling T (2004) Characterization of smooth muscle cell and pericyte differentiation in the rat retina in vivo. Invest Ophthalmol Vis Sci 45(8): 2795–806. 15277506 10.1167/iovs.03-1312

[36] Pournaras CJ, Rungger-Brandle E, Riva CE, Hardarson SH, Stefansson E (2008) Regulation of retinal blood flow in health and disease. Prog Retin Eye Res 27(3): 284–330. 18448380 10.1016/j.preteyeres.2008.02.002

[37] Puro DG (2012) Retinovascular physiology and pathophysiology: new experimental approach/new insights. Prog Retin Eye Res 31(3): 258–70. 22333041 10.1016/j.preteyeres.2012.01.001 PMC3334444

