# Supplementary figures and images for: Automated Detection and Measurement of Isolated Retinal Arterioles by a Combination of Edge Enhancement and Cost Analysis
Source: PLoS One. 2014 Mar 13;9(3):e91791. doi: 10.1371/journal.pone.0091791 (PMC3953588; doi:10.1371/journal.pone.0091791)

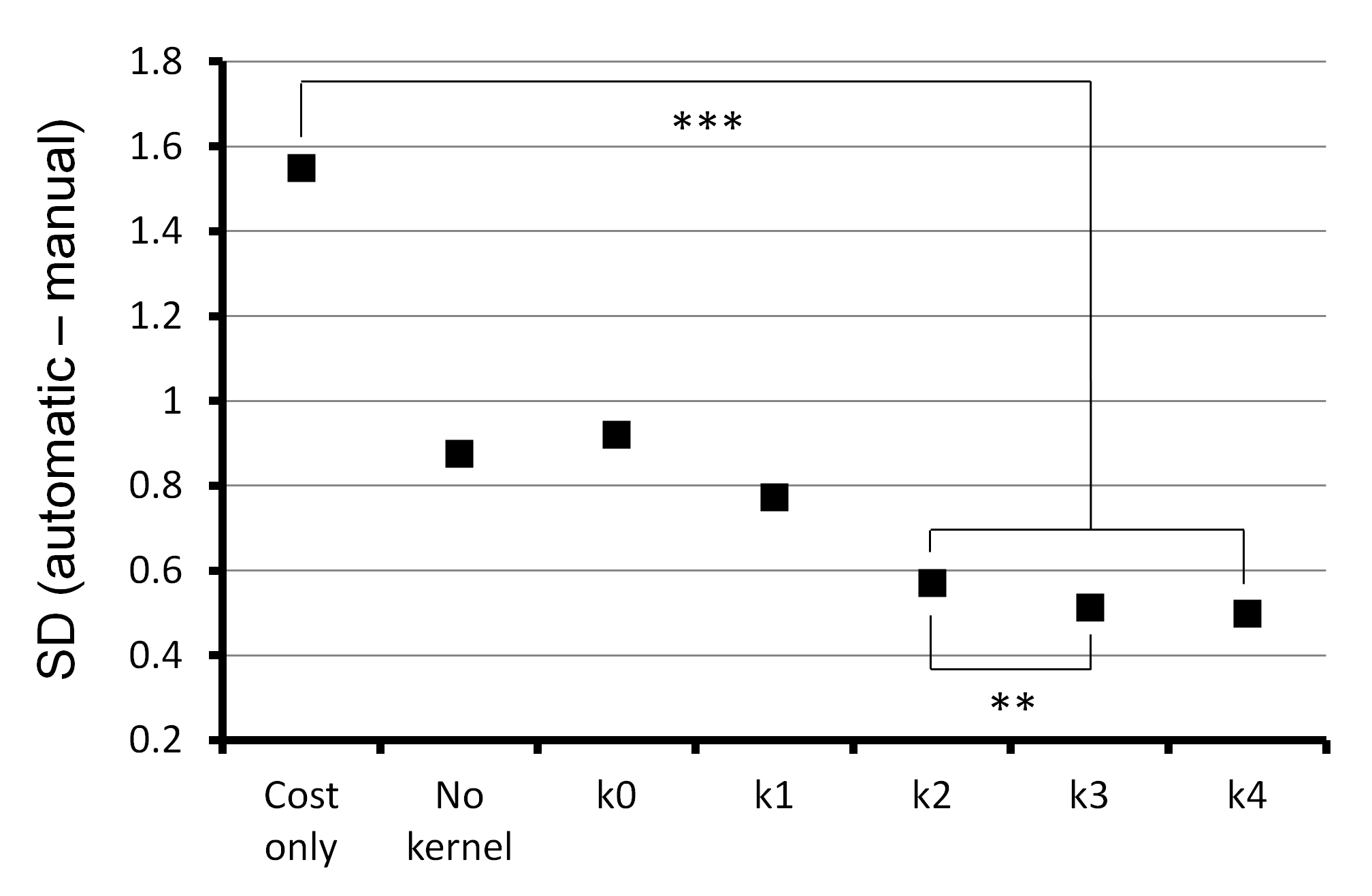

Supplement: Figure S1 — Measurement errors for different parameters in the algorithm. The error is shown as the SD of the difference between automatic and manual measurements in each case. The automatic values were obtained by the algorithm with either, only cost function and no further correction, or cost function and 6 different corrections: no kernel (using the original image with fitting parameters as outlined in Fig. 2 but without edge enhancement), k0 (using edge enhancement with kernels top = [−1, 0, 1] and bottom = [1,0,−1]), k1 (top = [−1, 1, 1] and bottom = [1,1,−1]), k2 (top = [−2, 1, 2] and bottom = [2,1,−2]), k3 (top = [−3, 1, 3] and bottom = [3,1,−3]), and k4 (top = [−4, 1, 4] and bottom = [4,1,−4]). The measurements were carried out in 101 of the 102 images in the dataset (image #8 was left out of the analysis because the detection failed with no kernel and with k1). The plot shows a significant improvement (decrease) in the values of the measurement errors with increasing correction (P<0.001 between only cost function and the last 3 kernels, k2, k3 and k4), up to a point where no further significant improvement was detected (P<0.01 between k2 and k3; P>0.05 between k3 and k4). Thus, k3 was selected as the default kernel for the algorithm in this study. Significance was estimated using One-way Repeated Measures ANOVA with Newman-Keuls Multiple Comparison post-hoc test. (TIF) [file pone.0091791.s001.tif]

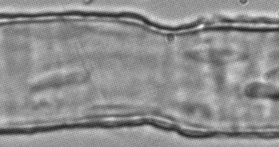

Supplement: Dataset S1 — First set of 51 images of retinal arterioles used for the analysis. (ZIP) [file pone.0091791.s003.zip › Dataset S1/1.tif]
