# Supplementary material for: Automated Detection and Measurement of Isolated Retinal Arterioles by a Combination of Edge Enhancement and Cost Analysis
Source: PLoS One. 2014 Mar 13;9(3):e91791. doi: 10.1371/journal.pone.0091791 (PMC3953588; doi:10.1371/journal.pone.0091791)
Supplement: File S1 — MyoTracker’s code and manual. The code folder contains both the source code (JAVA File) and the Executable Jar File for the plug-in. This allows the execution of the software within the FIJI (Image-J) environment, as explained in the manual. Copyright and GNU General Public License files are also included in this folder. The documentation folder includes the manual with instructions regarding the installation and use of the software. (ZIP) [file pone.0091791.s002.zip › File S1/MyoTracker documentation/MyoTracker documentation.pdf]

MyoTracker

José A. Fernández

Developed at:

Centre for Experimental Medicine, The Queen's University of Belfast, UK

Contents

1. Introduction

2. Getting started

    2.1. Main idea behind the algorithm

    2.2. Installing and running MyoTracker

    2.3. User interface and parameters

    2.4. Presentation of the results

3. Working examples

    3.1. Analysis of a simple individual vessel

    3.2. Analysis of a complex individual vessel

    3.3. Analysis of a video

4. Structure and components of the algorithm

1. Introduction

Myotracker is software developed to assist with the analysis of images and videos taken from myography experiments on isolated vessels. The algorithm is based on a combination of cost analysis and edge enhancement, to avoid pitfalls related to the overreliance on thresholding methods. The program is easy to run and is fully automatic, meaning that it does not initially require the user to make any manual measurements on the vessel under analysis, nor does it require setting any parameters.

It has been tested using images from different kinds of vessels – arteries, arterioles, venules and capillaries – of sizes ranging from ~5 to ~200μm in different inclinations, positions and intensity levels. Different options and parameters, however, are available to improve on the initial measurements provided. The program has been written as an ImageJ plug-in and it is freely available for use and further development.

This plug-in is made freely available, open-source and has been distributed under the terms of the GNU General Public License as published by the Free Software Foundation, either version 3 of the License, or (at your option) any later version.

2. Getting started

2.1. Main idea behind the algorithm

Once the image of a horizontally oriented vessel is opened, the software automatically detects the walls of the vessel, draws two lines along the middle of the walls, and measures the diameter of the vessel by averaging the distances between the two lines. The core of the algorithm is the cost function. Given some initially estimated start and end points (left and right sides of the vessel, respectively), the cost function draws a line from left to right making sure that each pixel in the line corresponds to a value that minimizes the combination between its distance to the estimated start and end points and the intensity gradient around it. To improve the performance of the cost function, edge enhancement is initially applied to the image. In this way, the adluminal and abluminal edges of the vessel walls are enhanced, thus increasing the intensity gradient subsequently used in the cost function.

2.2. Installing and running Myotracker

To install Myotracker, FIJI (FIJI Is Just ImageJ) is needed. A version equal or higher than 1.47o should first be installed in the computer. This can be done by opening the FIJI website:

http://fiji.sc/Fiji

and following the installation instructions after clicking on Download FIJI now.

After downloading FIJI, the application can be run directly from the folder obtained by double clicking on the FIJI application icon. When this is done, a small menu opens:

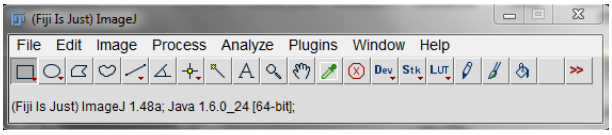

Figure 1. Running FIJI application on the computer

After installing ImageJ, the version obtained should be the latest available. If this is not the case, the software can be updated by clicking on Help -> Update ImageJ...

To install Myotracker, just drag and drop the Myotracker executable JAR file into the FIJI menu window. Then, click on Help -> Refresh Menus. Myotracker should now be part of the Plugins drop down menu. This plug-in works for all versions of ImageJ later than 1.48a, which is the version that was used to generate the results in the accompanying paper.

To run Myotracker, open an image of interest containing a horizontally oriented vessel, and click on Plugins -> Myotracker. The following menu opens:

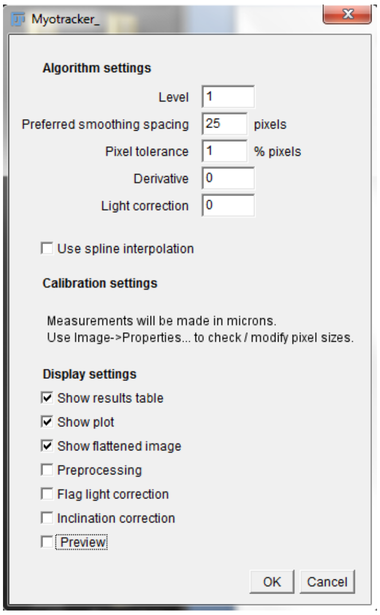

Figure 2. Running Myotracker application from FIJI Plugins folder

Finally, click OK to run the plug-in on the opened image.

2.3. User interface and parameters

Figure 2 shows the main Myotracker menu dialog. The dialog allows the user to set several parameters and check boxes. These are explained below:

Level: Divides the original image into narrower sub-images of the same initial height on which the algorithm runs independently (ranges from 1 to 5).

Preferred smoothing spacing: Distance between the consecutive points used for the drawing of the lines along the walls of the vessels. The final number of points depends on the length of the vessel under analysis (default distance=25 pixels, up to a maximum of 40 points).

Pixel tolerance: Determines the maximum number of wrong pixels that can be allowed in the wall lines (percentage of overall length of the vessel; ranges from 1 to 50%).

Derivative: Modifies the initial image (or slice) used for detection by applying derivatives of the order indicated (defaulted to 0). This allows the user to check whether alternative lines can be detected when the ones obtained are not satisfactory. This option can be useful when images are particularly complex. The parameter only works when the option *Preprocessing* (an initial Gaussian filtering of the image) is checked.

Light correction: Attempts to correct the error produced when the walls of the vessel under analysis are substantially lighter than the rest of the image (e.g. when a change of focus causes the vessel walls to become very light in intensity). In these cases, the detected lines will occasionally be overestimated, as the detection might be shifted to darker regions around the abluminal edges of the walls, away from their middle. This parameter only works when the option *Flag light correction* is checked. When the flag is set, the program attempts to apply a correction automatically on all slices, using as a guide the numerical value introduced. This is done during the drawing of the middle lines (see Methods in related paper) and works by comparing the 2 lines detected per wall to determine whether they are consistently very close to each other (the measure of 'closeness' is provided by the numerical parameter). The comparisons take place at each of the points used to draw the middle lines. When there is evidence that these lines are very close to each other (either the 2 at the top wall or the 2 at the bottom wall), the correction is applied. If the number of points when this happens is more than half of all the points checked, the refinement of the final measurement lines is deactivated to make sure the corrected lines remain unmodified.

Use spline interpolation: Provides an alternative way to join the points of the lines drawn through the middle of the walls by using splines instead of straight lines.

Inclination correction: Provides an alternative way to measure the diameters when the vessels under analysis are not horizontally oriented in the image (e.g. when they are inclined or twisted), as shown in the image below (Fig.3):

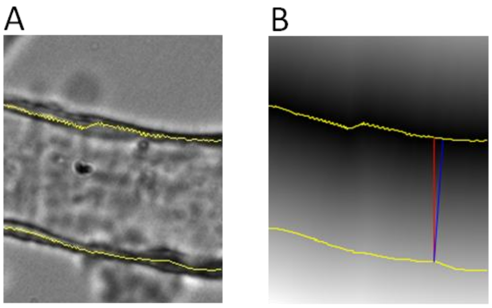

Figure 3. Running inclination correction A) Vessel with superimposed detected lines. B) Distance transform of the top wall line on the initial image. The red line shows the vertical distance between a pixel in the bottom line and the top line. The blue line shows the minimum distance between them (as given by the distance transform value.

When this option is checked, instead of measuring the vertical distance between the detected top and bottom lines (Fig. 3 B, red line), the shortest distance between the lines is calculated pixel by pixel (Fig. 3 B, blue line) and the final average is provided. This is accomplished by running the distance transform on a binary image containing one of the lines, and getting the pixel values in the distance transformed image for each of the coordinates of the other line (Fig. 3 B).

Preview: Draws the detected lines on top of the opened image (or the selected slice) for inspection by the user before actually running the program.

2.4. Presentation of the results

Myotracker runs independently on each image/slice. After the last slice is analysed, the program generates output values. A combination of several outputs can be provided:

Results table: Gives the mean diameter of the vessel for the image/slice analysed, as well as the minimum, the maximum and the median values of all the diameters within that slice. It also indicates the file and slice number and the units of the output measurements. The units value can be modified using the Image -> Properties dialog in the ImageJ menu.

Plot: Gives two lines showing, (i) the diameters measured per slice (gray line), and (ii) a running average of these diameters with a bin of 11 slices (black line).

Flattened image: Gives a video (or a single image) containing the analysed images with a flattened overlay of the detected lines on top of the vessel per slice.

3. Working examples

3.1. Analysis of a simple individual vessel

To analyse a vessel, several steps can be followed:

A. Open an image of interest using the ImageJ menu (File -> Open):

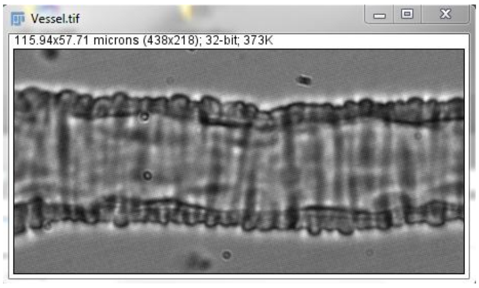

Figure 4. Image of a vessel opened with FIJI

B. Click on Preview to check whether the detected lines are suitable:

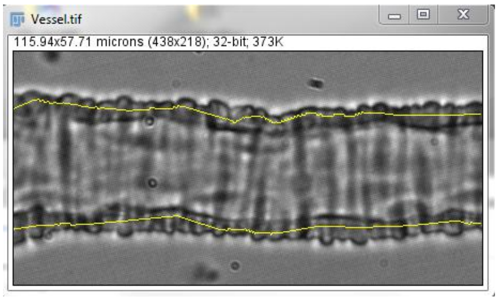

Figure 5. Detected lines superimposed on the vessel after checking Preview

C. If the lines are suitable, click OK. Several outputs are provided (if selected):

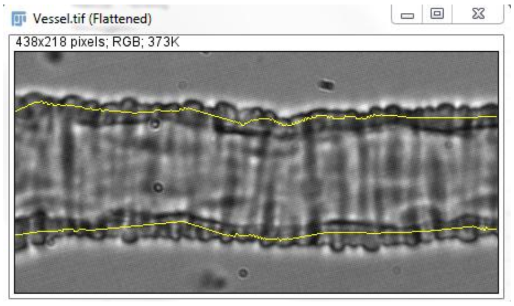

Figure 6. Flattened image containing the initial vessel and the detected lines

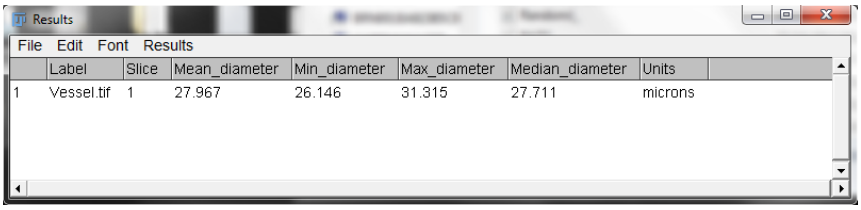

	Label	Slice	Mean_diameter	Min_diameter	Max_diameter	Median_diameter	Units
1	Vessel.tif	1	27.967	26.146	31.315	27.711	microns

Figure 7. Results table containing different values of interest

3.2. Analysis of a complex individual vessel

When the vessel under analysis is more complex than shown above, several parameters are provided to improve on the line detection. Although the steps will vary in each case depending on the particular image under analysis, the steps of analysis can be as follows:

A. Open an image of interest using the ImageJ menu (File -> Open):

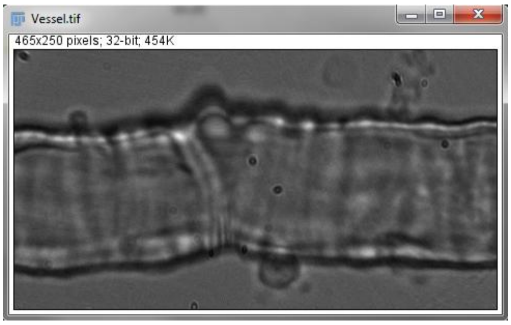

Figure 8. Image of another vessel opened with FIJI

B. Click on Preview to check whether the detected lines are suitable:

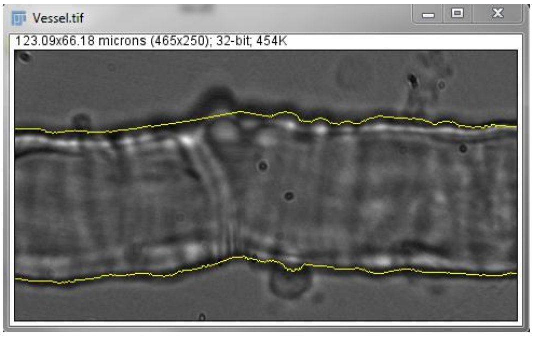

Figure 9. Detected lines superimposed on the vessel after checking Preview

The estimated lines shown above seem to overestimate the middle of the walls. This is more clearly seen when comparing these lines with the manual measurements provided by a user:

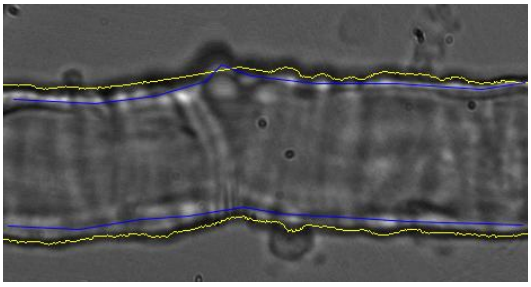

Figure 10. Comparison of the detected lines superimposed on the vessel (yellow lines) and manual lines provided by a user (blue lines)

C. To improve on the detected lines, we can use the *Light correction* flag provided in the initial dialog. By checking this box and setting a value of 9 (equivalent to 2.4μm, estimated by the manual measurement of the wall-to-lumen distance), the overestimation is reduced:

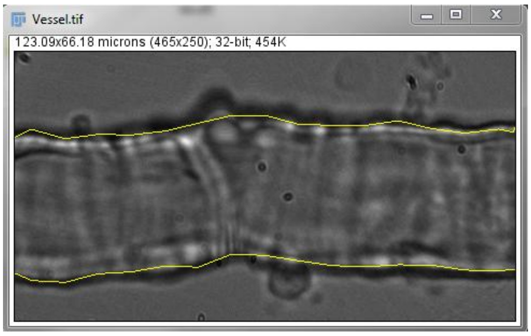

Figure 11. Detected lines superimposed on the vessel with *Light Correction*=9

D. Further improvement can be obtained by checking the *Preprocessing* flag and setting a *Derivative* value of 5:

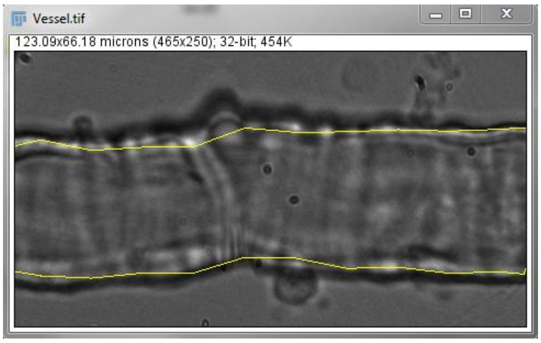

Figure 12. Detected lines superimposed on the vessel with *Light Correction*=9, *Preprocessing* and *Derivative*=5

E. The final results obtained after this analysis are as follows:

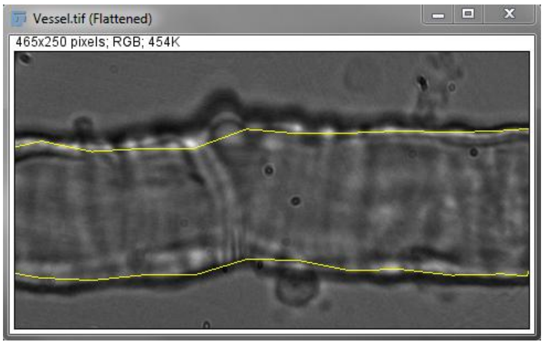

Figure 13. Flattened image containing the initial vessel and the detected lines

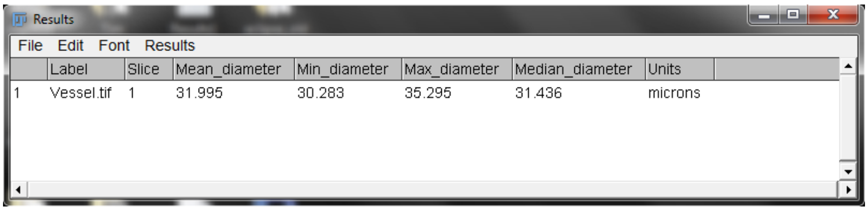

	Label	Slice	Mean_diameter	Min_diameter	Max_diameter	Median_diameter	Units
1	Vessel.tif	1	31.995	30.283	35.295	31.436	microns

Figure 14. Results table containing different values of interest

As can be seen in the Results table, the value provided for the diameter in this last case is just under 32μm. This is very close to the 31.35μm obtained from the manual measurement shown above (Fig. 10).

3.3. Analysis of a video

Videos are analysed in a similar way to individual images:

A. Open a video of interest using the ImageJ menu (File -> Open):

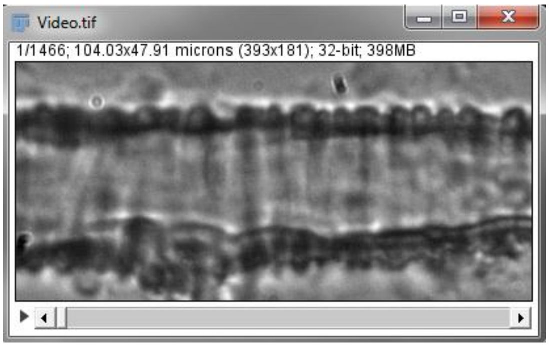

Figure 15. Video of vessel experiment opened with FIJI

B. Click on Preview to check whether the detected lines are suitable:

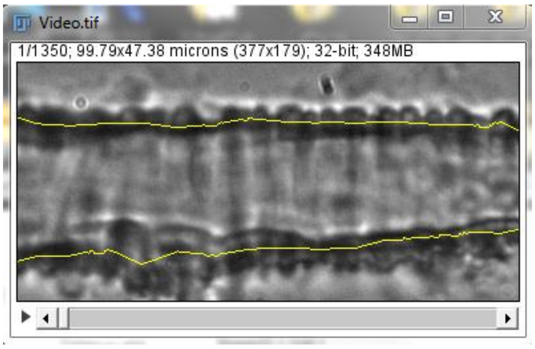

Figure 16. Detected lines superimposed on the vessel after checking Preview

The *Preview* option can be run on slices different from the first one. To achieve this, move to the slice of interest before opening the Myotracker dialog.

C. If the lines are suitable, click OK. The program will run on all slices from beginning to end. An extra option, *Show progress frame by frame (slower)*, appears in the initial dialog when running on videos:

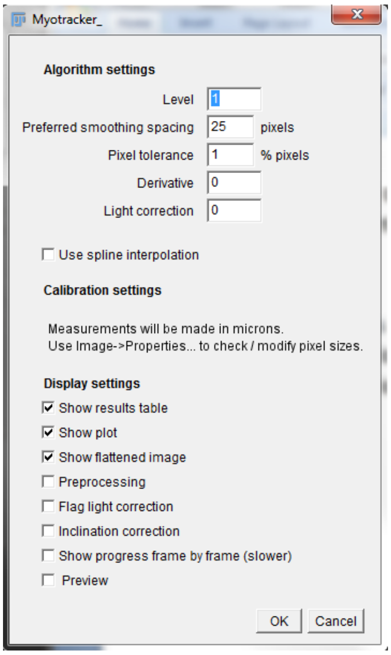

Figure 17. Main Myotracker initial dialog for processing videos

This option allows the user to see the detected lines slice per slice as the program runs. As the label indicates, the processing is lower when this option is checked. After running, several outputs are provided (if selected):

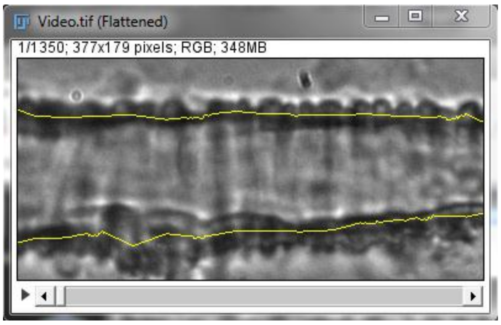

Figure 18. Flattened video containing the initial vessel and the detected lines per slice

	Label	Slice	Mean_diameter	Min_diameter	Max_diameter	Median_diameter	Units
1	Video.tif	1	24.780	19.912	28.588	25.265	microns
2	Video.tif	2	24.792	19.226	28.430	25.233	microns
3	Video.tif	3	24.574	19.238	27.652	25.145	microns
4	Video.tif	4	24.753	19.319	28.324	25.314	microns
5	Video.tif	5	24.802	20.096	28.324	25.399	microns
6	Video.tif	6	24.742	20.531	27.911	25.421	microns
7	Video.tif	7	24.805	20.530	29.413	25.168	microns
8	Video.tif	8	24.776	17.665	29.029	25.099	microns
9	Video.tif	9	24.732	20.148	28.721	25.100	microns
10	Video.tif	10	24.658	18.926	28.588	25.002	microns
11	Video.tif	11	24.530	18.780	28.324	24.786	microns
12	Video.tif	12	24.540	19.175	28.166	24.977	microns
13	Video.tif	13	24.660	20.131	28.853	24.989	microns
14	Video.tif	14	24.618	18.796	28.400	24.936	microns
15	Video.tif	15	24.733	18.887	29.832	25.161	microns
16	Video.tif	16	24.482	18.959	27.886	24.757	microns
17	Video.tif	17	24.628	18.331	27.911	25.078	microns
18	Video.tif	18	24.643	18.385	27.860	25.266	microns
19	Video.tif	19	24.765	19.291	28.846	25.074	microns
20	Video.tif	20	24.673	18.433	27.850	25.066	microns
21	Video.tif	21	24.641	18.481	28.985	25.140	microns
22	Video.tif	22	24.692	18.362	28.721	25.205	microns
23	Video.tif	23	24.773	20.515	28.985	25.231	microns
24	Video.tif	24	24.734	18.960	28.059	25.134	microns
25	Video.tif	25	24.771	19.695	27.687	25.188	microns
26	Video.tif	26	24.707	18.735	27.438	25.178	microns
27	Video.tif	27	24.743	19.205	27.545	25.346	microns
28	Video.tif	28	24.676	19.184	27.641	25.125	microns

Figure 19. Results table containing different values of interest per slice

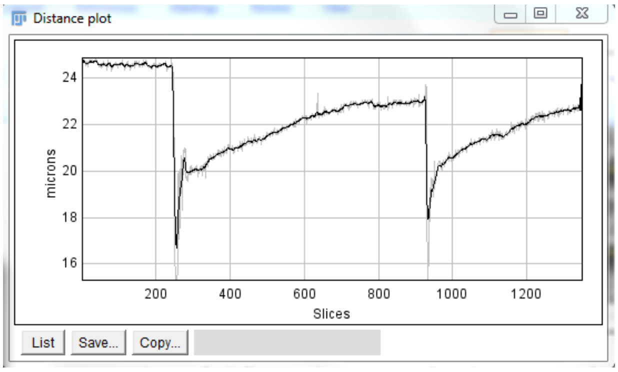

Figure 20. Plot containing the raw value of the diameters measured per slice (grey lines) and a running average (bin=11) superimposed on the raw values (black line).

4. Structure and components of the algorithm

The Myotracker algorithm is implemented as a public class using the Java language. This class implements both an ExtendedPlugInFilter and a DialogListener. The program is developed using an object oriented programming approach. There are two main classes in the program, the Vessel class and the Myotracker class. The first one contains all the functionality (variables and methods) directly related to the Vessel itself, whereas the second contains the functions related to the main running of the program.

A. Vessel class: This class contains three attributes:

top_border, bottom_border: Private objects of type FloatPolygon, each containing the x- and y-values of the corresponding top and bottom lines along the walls of the vessel.

slice: Integer variable indicating the current slice.

The class also contains some getter and setter methods to manage the private attributes and return the diameters:

get_top_ROI, get_bottom_ROI: Return top or bottom borders as PolygonRoi objects.

get_top_border_polygon, get_bottom_border_polygon: Return top or bottom borders as FloatPolygon objects.

reset_vessel: Set top_border and bottom_border FloatPolygon.npoints values to 0.

get_slice, set_slice: Return or set the value of the slice attribute.

contains_valid_diameters: Check that top_border and bottom_border values are filled and that the number of values in each line are the same.

get_diameters: Return an array of Double values containing the difference between top and bottom y-values.

get_corrected_diameters: Return an array of Double values containing the minimum distance between each of the bottom values and the top values.

get_units: Returns a String value containing the units specified in the Image -> Properties setting (or "pixels" as default).

B. Myotracker class: This class contains all the attributes necessary to run the program and store the parameters passed to the main initial dialog (i.e. the parameter values to be set by the user and the flags to be checked or unchecked). It also contains several methods to handle the processing of the images. They can be divided into the following categories:

B.1. First layer of methods: Overall running of the algorithm

setup: Initialises the parameters and options, and provides functionality to generate output results when the final slice is run.

run: Processes each image/slice independently of the rest.

B.2. Second layer of methods: Iterative processing

measure_vessel: Handles the iterative processing of the program by fitting the level, smoother and pixel_tolerance parameters.

get_window: Handles the fitting of the window parameter during iterations.

B.3. Third layer of methods: Core functionality

convolve_duplicate: Returns a convolved image according to some kernel values. This function is used to enhance the edges of the original vessel walls.

draw_border: Draws lines making use of the cost function. This is used to draw lines along the adluminal and abluminal edges of the top and bottom walls of the vessel using cost function analysis.

cost: Cost function returning a value for a particular pixel, obtained by balancing out its distance to estimated start and end points, and its position within an intensity gradient.

get_middles: Finds middle points between two lines passed as parameters. This function provides the points over which the middle lines on each wall will be drawn.

make_binary: Creates a binary image. This is used for validation of the drawn lines.

line_invalid: Checks whether the detected lines fall within the allowed section of the binary image used for validation.

refine_binary_for_localization: Modifies the binary image used for validation to improve the drawing of the lines (i.e. to adjust the lines to the walls of the vessel).

refine_border: Shifts the estimated lines using the refined binary image as a guide.

B.4. Fourth layer of methods: Output generation

create_flattened_image: Generates a flattened version of the original image/video with the detected lines superimposed per slice.

do_plot: Generates a plot with the raw average of the vessel diameters per slice (in grey) and a moving average of the diameters superimposed on top of the raw values (in black).

B.5. Fifth layer of methods: Dialog control

showDialog: Controls the initial dialog as well as the setting and storing of the different parameters and options.

dialogItemChanged: Event-driven section of the dialog handling user choices.

B.6. Sixth layer of methods: Helper functions

preprocess: Applies a Gaussian filter to an image when Preprocessing checked.

get_flags: Returns a set of flag options corresponding to the parameter *show_progressing*.

crop_image: Returns a cropped version of an image.

moving_average: Returns an array of Double values obtained by a moving average of a bin value passed as a parameter.

get_min_and_max: Returns the min and max values of an array of Double values.

update_path: Updates the FloatPolygon of interest (usually one of the Vessel attributes).

create_split_regions: Splits an initial image into a series of narrower sub-images according to a level value.

m_interp: Interpolates between multiple points.

interp_linear: Interpolates between two points.

resize: Resizes an array.

mean_pixel_column: Returns the mean of intensities between two points in the same column.

dist: Calculates the distance between two points.

get_initial_y_estimate: Provides an estimate for start and end points. These are used a initial values in the cost function.

array_mean: Gives the mean of an array.

next_pixel_lower: Compares two adjacent pixels.

calculate_interp_factor: Sets the interpolation factor from smoother.

calculate_max_wrong_pixels: Sets the maximum number of wrong pixels from pixel tolerance.

get_last_x: Returns the last x-value of a FloatPolygon.

get_last_y: Returns the last y-value of a FloatPolygon.
